# Ipilimumab and nivolumab plus radioembolization as salvage therapy for atezolizumab and bevacizumab refractory hepatocellular carcinoma resulting in complete pathologic response

**DOI:** 10.1016/j.radcr.2024.07.157

**Published:** 2024-08-18

**Authors:** Claudia R. Silver, Cynthia De la Garza-Ramos, John A. Stauffer, Umair Majeed, Jianfeng Wang, Beau B. Toskich

**Affiliations:** a Florida State University College of Medicine, Tallahassee, Florida; b Division of Interventional Radiology, Mayo Clinic Florida, Jacksonville, Florida; c Department of Surgery, Mayo Clinic Florida, Jacksonville, Florida; d Department of Hematology Oncology, Mayo Clinic Florida, Jacksonville, Florida; e Department of Hematology Oncology, Pearlman Comprehensive Cancer Center at South Georgia Medical Center, Valdosta, Georgia

**Keywords:** Radioembolization, Y90, Hepatocellular carcinoma, Immunotherapy, PD-L1/CTLA-4 blockade, Combination therapies

## Abstract

Unresectable hepatocellular carcinoma unresponsive to first-line immunotherapy has a poor prognosis with modest response to tyrosine kinase inhibitors in the second line. In these patients, the benefit of local therapy with immunotherapy rechallenge is unknown. Radioembolization is a guideline-supported locoregional therapy for HCC that has shown the potential for synergy in combination with immunotherapy. This report describes a patient with veno-invasive HCC and extrahepatic invasion of the right kidney which progressed on atezolizumab and bevacizumab and was subsequently downstaged to resection with ipilimumab and nivolumab plus radioembolization yielding a complete pathologic response. The patient is currently more than 2 years since diagnosis without evidence of disease recurrence.

## Introduction

Hepatocellular carcinoma (HCC) is the most common primary liver cancer [1] and most patients present with unresectable disease. Patients with metastatic HCC often have a poor prognosis with a 5-year survival rate of 2% [2]. Anti-programmed cell death-1 ligand-1 (PD-L1) antibody (atezolizumab) and anti-vascular endothelial growth factor (VEGF) antibody (bevacizumab) are considered standard-of-care, first-line, systemic agents for advanced HCC, yet yield a 27.3% objective response rate and 13.9-month survival rate in patients with a baseline AFP ≥ 400 ng/mL [3]. Re-challenging HCC with alternate immunotherapy regimens and local therapy after initial immunotherapy failure is not well understood [4]. Combination therapies may potentially offer additive or synergistic effects that could benefit patients with challenging disease presentations. Transasrterial radioembolization may result in release of tumor antigens and immune activation that may increase the likelihood of response to immunotherapy [5].

This report presents a case of advanced HCC with macrovascular invasion (MVI) and extrahepatic spread (EHS) unresponsive to first-line atezolizumab and bevacizumab that was subsequently downstaged to resection with a combination of anti-CTLA-4 (ipilimumab) and anti-PD-1 (nivolumab) checkpoint inhibitors plus radioembolization resulting in complete pathologic response.

## Case report

**Fig. 1:**
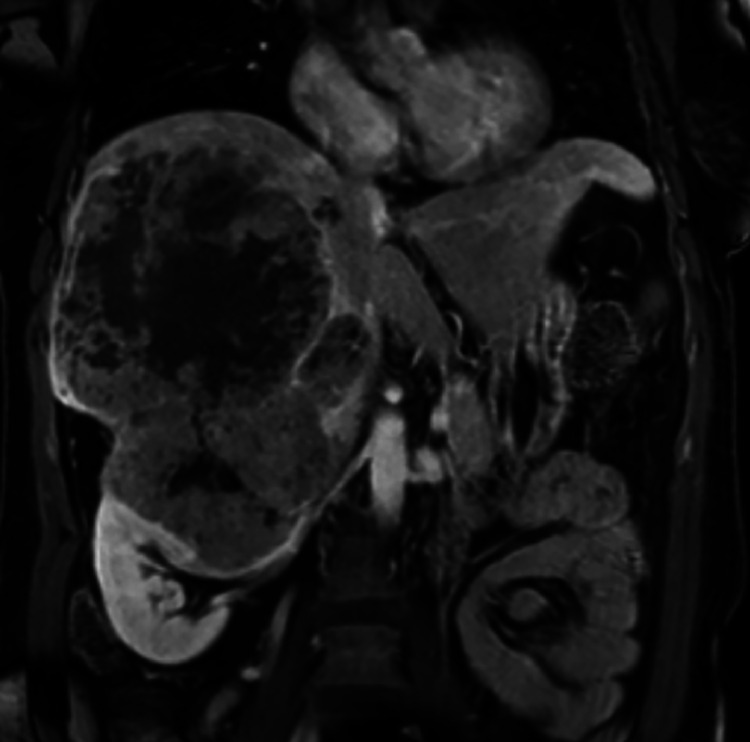
Initial T1-weighted postcontrast MRI demonstrated a 15 cm centrally necrotic HCC within the right hepatic lobe with invasion of the right upper renal pole and suspected invasion of the right adrenal gland.

A 55-year-old woman presented to a liver transplant center with a 15 cm right hepatic lobe HCC with invasion of the superior right renal pole and an alpha-fetoprotein (AFP) level of 169,343 ng/mL (Fig. 1). The patient had a history of hyperlipidemia and metabolic-associated steatotic liver disease without cirrhosis, with Child-Pugh A and ALBI 1 liver function, and an Eastern Cooperative Oncology Group (ECOG) score of 0.

**Fig. 2:**
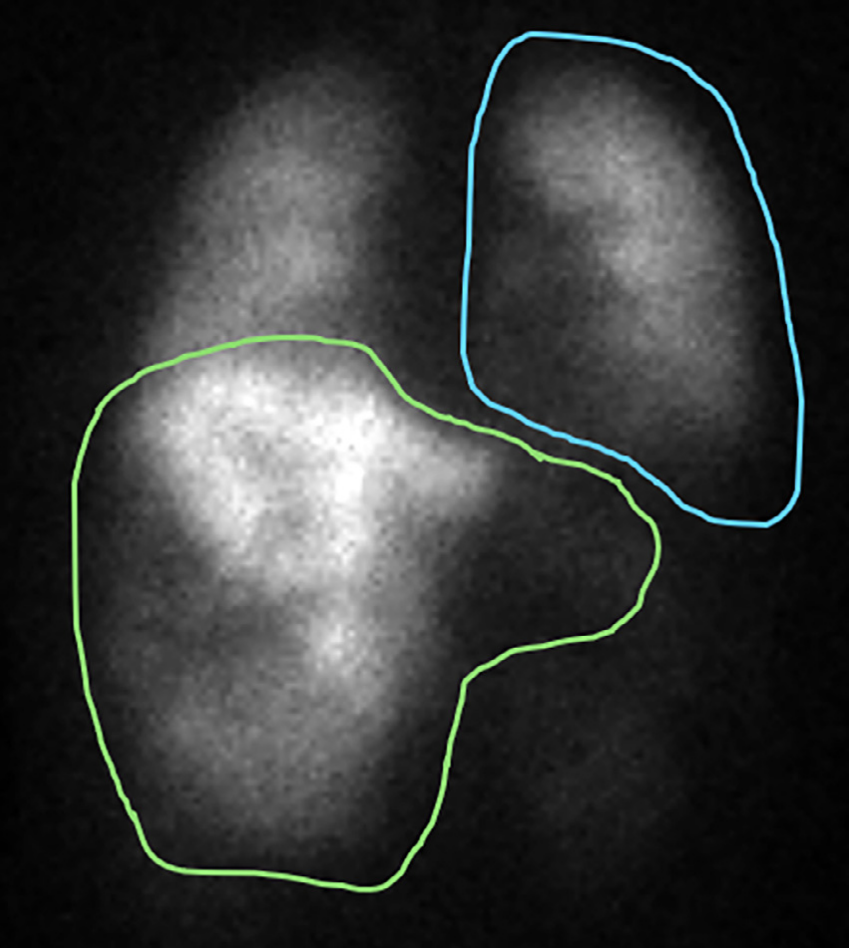
Planar scintigraphy after initial mapping with 99Tc-MAA demonstrated substantial radiotracer uptake within the liver as well as the lungs. The calculated LSF was 25%. Green: Liver. Blue: Lung.

Following a evaluation at a multidisciplinary tumor board, the patient was referred to Interventional Radiology to assess her candidacy for neoadjuvant radiation lobectomy as a means of downstaging to surgical resection. Transarterial technetium macroaggregated albumin (99Tc-MAA) infusion during mapping angiography of the tumor-supplying arteries revealed a lung shunt fraction (LSF) of 25% (Fig. 2), precluding the patient from definitive dose radioembolization (>205 Gy) given the increased risk of radiation pneumonitis [6].

The patient was subsequently treated with palliative-intent atezolizumab and bevacizumab. At 6-month follow-up, the AFP had increased to 448,900 ng/mL and the right hepatic lobe HCC increased in diameter to 19 cm, with tumor invasion of the IVC. The patient's performance had deteriorated to an ECOG score of 2. Given disease progression, the decision was made to rechallenge the patient with doublet immunotherapy combination and administer a palliative dose of radioembolization. Five days after initiating ipilimumab and nivolumab, the patient underwent radioembolization with a right lobar tumor multicompartment dose of 58 Gy using Yttrium-90 (Y90)-containing glass microspheres (Therasphere, Boston Scientific, Malborough, MA) with 9-day decay. The estimated lung dose was 19 Gy.

**Fig. 3:**
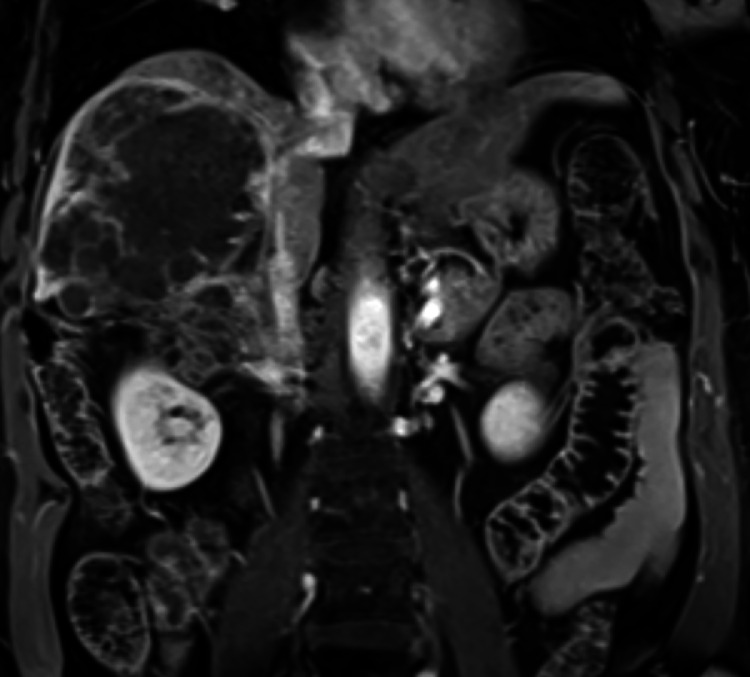
T1-weighted postcontrast MRI 4 months after radioembolization plus ipilimumab and nivolumab demonstrated positive treatment response without concerning enhancement within the right hepatic lobe HCC, now decreased in size with a maximum diameter of 11.5 cm.

**Fig. 4:**
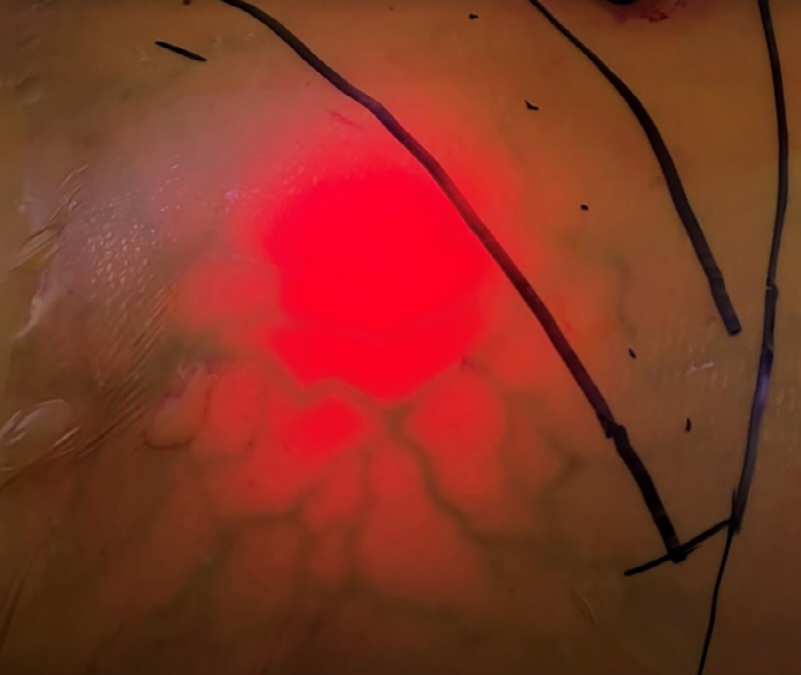
The abdominal venous collaterals were mapped out with laparoscopic transillumination to avoid transection and preserve lower extremity drainage.

**Fig. 5:**
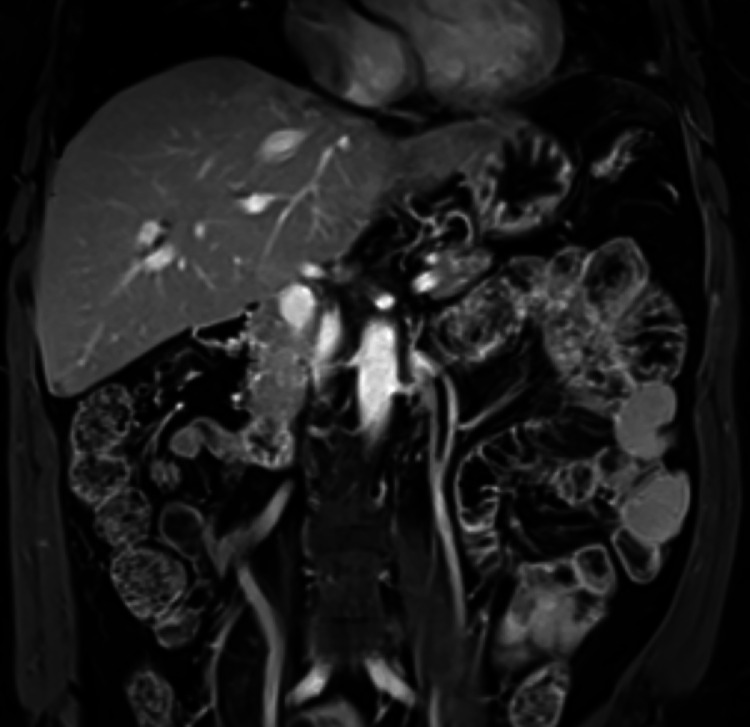
Most recent follow-up T1-weighted post-contrast MRI obtained 16 months after en-bloc resection demonstrated complete response without evidence of residual or recurrent disease.

Four months later, the AFP had decreased to 64 ng/mL and the patient's performance had improved to ECOG 0. A contrast-enhanced multiphase abdominal MRI demonstrated no residual concerning enhancement within the tumor (Fig. 3). The IVC invasive tumor moiety had retracted and resulted in caval occlusion with development of multiple abdominal wall venous collaterals that were mapped out with laparoscopic transillumination (Fig. 4). The patient subsequently underwent a successful open, en-bloc right hepatectomy, right nephrectomy, and right adrenalectomy with local lymph node resection. Surgical pathology revealed no residual viable tumor and resected lymph nodes were negative for malignancy. Postoperatively, the patient experienced a mild increase in her baseline creatinine but was otherwise without complication. The patient remains on maintenance nivolumab 3 mg/kg every 2 weeks and has had no evidence of disease recurrence 16 months after resection (Fig. 5).

## Discussion

Immunotherapy-based systemic therapy is the first-line treatment for patients with advanced HCC [7]. As most patients eventually progress, a common strategy is to treat with second-line tyrosine kinase inhibitors despite poor response rates. Little is known about salvaging initial immunotherapy nonresponders with an alternative combination of immunotherapy and local therapy.

In the presented case, a patient with HCC with MVI and EHS that progressed on first-line atezolizumab and bevacizumab was successfully downstaged to surgical resection with a combination of ipilimumab and nivolumab plus radioembolization. The patient's disease burden at the time of progression was characterized by a high AFP, which decreased from 448,900 ng/mL to 64 ng/mL 4 months after initiating this combination regimen, and later normalized to 1.0 ng/mL after resection. The patient did not experience major treatment-related adverse events and is currently without evidence of disease. This case illustrates the potential for downstaging advanced patients to curative intent treatments using a combination of local therapy and immunotherapy. Other cases of combination therapies leading to successful downstaging with complete pathologic response have been described; for example, a case of a patient with angioinvasive HCC who was successfully bridged to partial hepatectomy with combination nivolumab and radioembolization; however, the presented case adds additional value by exploring outcomes after failure following initial systemic therapy [8].

The phase III IMbrave150 trial established atezolizumab and bevacizumab as a first-line, standard-of-care, regimen for advanced HCC over sorafenib, but the overall response rate was 27.3%. Median overall survival (OS) for patients in this trial with an AFP ≥ 400 ng/mL, MVI, and EHS was 13.9 months, 14.2 months, and 17.8 months, respectively [3]. The phase III HIMALAYA trial compared tremelimumab and durvalumab (STRIDE) to sorenafib and durvalumab as monotherapies and established immunotherapy doublets as an effective first-line regimen. The reported death rate amongst patients given STRIDE with an AFP ≥ 400 ng/mL, MVI, and EHS was 75.5%, 78.6, and 75.6%, respectively. The same parameters were evaluated for those treated with sorenafib and death rates were 87.3%, 87.0%, and 81.8%, respectively [9]. Most recently, the phase III CheckMate-9DW trial compared the systemic regimen reported in this case (ipilimumab and nivolumab) to sorenafib or lenvatinib monotherapies as a first-line agent for advanced HCC (NCT04039607). This trial met its primary endpoint of improved OS compared to sorenafib and levatinib but is pending peer review [10].

The phase II DOSISPHERE-01 trial investigated outcomes for patients with unresectable, locally advanced HCC who received radioembolization using personalized dosimetry (PDA) or standardized dosimetry (SDA) and revealed a 24.8-month OS with PDA compared to a 10.7-month OS for SDA [6]. There is interest in augmenting these outcomes with a combination of radioembolization plus immunotherapy, particularly in patients who are not candidates for definitive radioembolization dosimetry, as in the current case. The recent positive phase III EMERALD-1 and IMbrave050 trials demonstrated that combination local and immunotherapy-based systemic therapy resulted in a significant improvement in PFS when compared to local therapy alone [11,12]. Alternatively, the benefit of adding local therapy to immunotherapy compared to immunotherapy alone remains unknown. Yeo et al. [5] conducted a retrospective National Cancer Database study assessing the combination of radioembolization and immunotherapy and reported a median OS of 19.8 months compared to 9.5 months for those who received immunotherapy alone. Current trials underway include EMERALD-90 and ROWAN, which will investigate patients with unresectable HCC receiving radioembolization in combination with subsequent durvalumab plus bevacizumab and durvalumab plus tremelimumab, respectively (NCT06040099, NCT05063565) [13,14].

The optimal dosimetry and sequencing of radioembolization and immunotherapy remain under active investigation. While ablative and personalized radioembolization dosimetry have shown improved outcomes in the definitive management of HCC, lower dose radioembolization that is spatially fractionated could theoretically stimulate the immune system [15]. Future applications could be tailored to oncologic intent, whether radioembolization is the definitive therapy and immunotherapy serves as an adjuvant modality, or vice versa. This will be difficult to study given the complexity of predicting the immune microenvironment and that synergistic interactions are rare. Nonetheless, future patients may be interested in salvage treatments with the potential for a positive outcome such as the presented case if they have low adverse events - even if there is a low probability of success. This concept of “lottery oncology” may require disruption of historical trial design to be successfully incorporated as a treatment paradigm and is only further complicated by the increasing permutations of novel immunotherapies [16].

## Conclusion

While systemic therapy for advanced HCC has shown significant improvement, most patients ultimately progress after first-line regimens. We present a case of HCC with MVI and EHS that progressed on atezolizumab and bevacizumab that was subsequently successfully downstaged to resection with complete pathologic response using a combination of ipilimumab and nivolumab plus radioembolization. The combination of immunotherapy and radioembolization in this population may serve as a bridge to curative treatment for refractory HCC and warrants further investigation.

## Patient consent

The patient's consent for the use of de-identified medical information for education and research purposes was obtained.
